# Direct Activation of RhoA by Reactive Oxygen Species Requires a Redox-Sensitive Motif

**DOI:** 10.1371/journal.pone.0008045

**Published:** 2009-11-26

**Authors:** Amir Aghajanian, Erika S. Wittchen, Sharon L. Campbell, Keith Burridge

**Affiliations:** 1 Department of Cell and Developmental Biology, University of North Carolina at Chapel Hill, Lineberger Comprehensive Cancer Center, Chapel Hill, North Carolina, United States of America; 2 Department of Biochemistry and Biophysics, University of North Carolina at Chapel Hill, Lineberger Comprehensive Cancer Center, Chapel Hill, North Carolina, United States of America; 3 UNC McAllister Heart Institute, University of North Carolina at Chapel Hill, Chapel Hill, North Carolina, United States of America; University of Massachusetts Amherst, United States of America

## Abstract

**Background:**

Rho family GTPases are critical regulators of the cytoskeleton and affect cell migration, cell-cell adhesion, and cell-matrix adhesion. As with all GTPases, their activity is determined by their guanine nucleotide-bound state. Understanding how Rho proteins are activated and inactivated has largely focused on regulatory proteins such as guanine nucleotide exchange factors (GEFs) and GTPase activating proteins (GAPs). However, recent *in vitro* studies have indicated that GTPases may also be directly regulated by redox agents. We hypothesized that this redox-based mechanism occurs in cells and affects cytoskeletal dynamics, and in this report we conclude this is indeed a novel mechanism of regulating the GTPase RhoA.

**Methodology/Principal Findings:**

In this report, we show that RhoA can be directly activated by reactive oxygen species (ROS) in cells, and that this requires two critical cysteine residues located in a unique redox-sensitive motif within the phosphoryl binding loop. First, we show that ROS can reversibly activate RhoA and induce stress fiber formation, a well characterized readout of RhoA activity. To determine the role of cysteine residues in this mechanism of regulation, we generated cysteine to alanine RhoA mutants. Mutation of these cysteines abolishes ROS-mediated activation and stress fiber formation, indicating that these residues are critical for redox-regulation of RhoA. Importantly, these mutants maintain the ability to be activated by GEFs.

**Conclusions/Significance:**

Our findings identify a novel mechanism for the regulation of RhoA in cells by ROS, which is independent of classical regulatory proteins. This mechanism of regulation may be particularly relevant in pathological conditions where ROS are generated and the cellular redox-balance altered, such as in asthma and ischemia-reperfusion injury.

## Introduction

Rho family GTPases serve as critical regulators of cell migration, cell-cell adhesion, and cell-matrix adhesion, by transmitting extracellular and intracellular signals to effectors that act on the cytoskeleton. The best characterized members of the Rho family of GTPases are RhoA, Rac1, and Cdc42; each of which is associated with unique phenotypes and functions [1], [2], [3]. As with all canonical GTPases, the activity of Rho GTPases is determined by their guanine nucleotide-bound state. Rho GTPases are activated by binding GTP, which causes a conformational change in the protein that greatly increases the affinity for downstream effector proteins. These effector proteins are components of signaling cascades which ultimately lead to modulation of cellular functions. Conversely, GDP-bound Rho GTPases are unable to bind effector proteins and are therefore inactive. The switching between “on” and “off” states is tightly controlled by regulatory proteins which interact with GTPases to regulate guanine nucleotide binding [4]. Guanine nucleotide exchange factors (GEFs) activate GTPases by promoting the dissociation of GDP to allow the binding of GTP, which is available in great excess over GDP levels in the cytoplasm. GTPase activating proteins (GAPs) promote the hydrolysis of GTP to GDP, preventing GTPase interaction with downstream effectors. GDP-dissociation inhibitors (GDIs) maintain the inactive state of the GTPase by preventing GDP-dissociation and membrane association [5]. All of these regulatory proteins are themselves affected by diverse upstream signals which serve to activate or inactivate Rho GTPase signaling pathways.

Yet another mechanism for regulating GTPase activity has been proposed that involves the action of redox agents, specifically reactive oxygen species (ROS) and reactive nitrogen species (RNS). ROS and RNS have been historically considered pathological agents which can react with and damage many biological macromolecules including DNA, proteins and lipids. However, there has been an increasing recognition that ROS and RNS can also function in cell signaling pathways [6], [7] in particular, those pathways involving phosphatases [8]. Most cell types produce ROS, either as byproducts of normal metabolism or by specific enzyme complexes. A major source of ROS in cells is the NADPH oxidase complex, which can be activated by Rac1 [9], [10], [11]. This generation of ROS by Rac1 has been implicated in the redox-mediated regulation of RhoA activity. Nimnual et al. described a novel mechanism in which downregulation of RhoA occurs via redox-mediated inactivation of the phosphatase LMW-PTP and subsequent elevation of p190 RhoGAP activity [12].

As well as affecting regulatory protein function, another body of work shows that at least *in vitro*, oxidizing agents can also directly regulate the activity of certain GTPases. Lander et al. were the first to show this with Ras; NO treatment of recombinant Ras increased the proportion of GTP-bound Ras and endogenous NO activated Ras in human T cells [13], [14]. Campbell's group went on to describe a radical-based mechanism for the stimulation of nucleotide exchange on Ras by NO [15]. Interestingly, Campbell's group also identified a distinct redox-active motif located in the phosphoryl-binding loop in another subset of GTPases, the Rho family (RhoA, Rac1, and Cdc42) [16]. They found that treatment of purified recombinant GTPase with superoxide anion radical or nitrogen dioxide radicals *in vitro* resulted in guanine nucleotide dissociation. The proposed mechanism involves electron transfer of a thiol radical intermediate to the guanine base, which disrupts interactions between the guanine nucleotide and the GTPase, resulting in guanine nucleotide dissociation. In the presence of radical quenching agents, the intermediates are reversed and guanine nucleotide association can occur. Under conditions where GTP is in excess, such as in the cell, the end result is exchange of GDP for GTP, thereby activating the GTPase. Based on these *in vitro* findings, we hypothesize that ROS can directly affect GTPase activity in cells by oxidative modification of the critical cysteine residues within the redox-active motif. This may be a novel mechanism of regulating GTPase signaling cascades, independent but parallel to classical regulation by GEFs and GAPs.

In this paper, we show that RhoA can be directly activated by ROS in cells and that ROS-mediated activation of RhoA can induce cytoskeletal rearrangement. By using cysteine to alanine mutants, we demonstrate that ROS-mediated activation of RhoA is dependent on cysteines 16 and 20. Our findings indicate that ROS-mediated RhoA activation is another potential regulatory mechanism in cells that can affect cytoskeletal dynamics.

## Results

### ROS Induces Reversible Activation of RhoA

**Figure 1:**
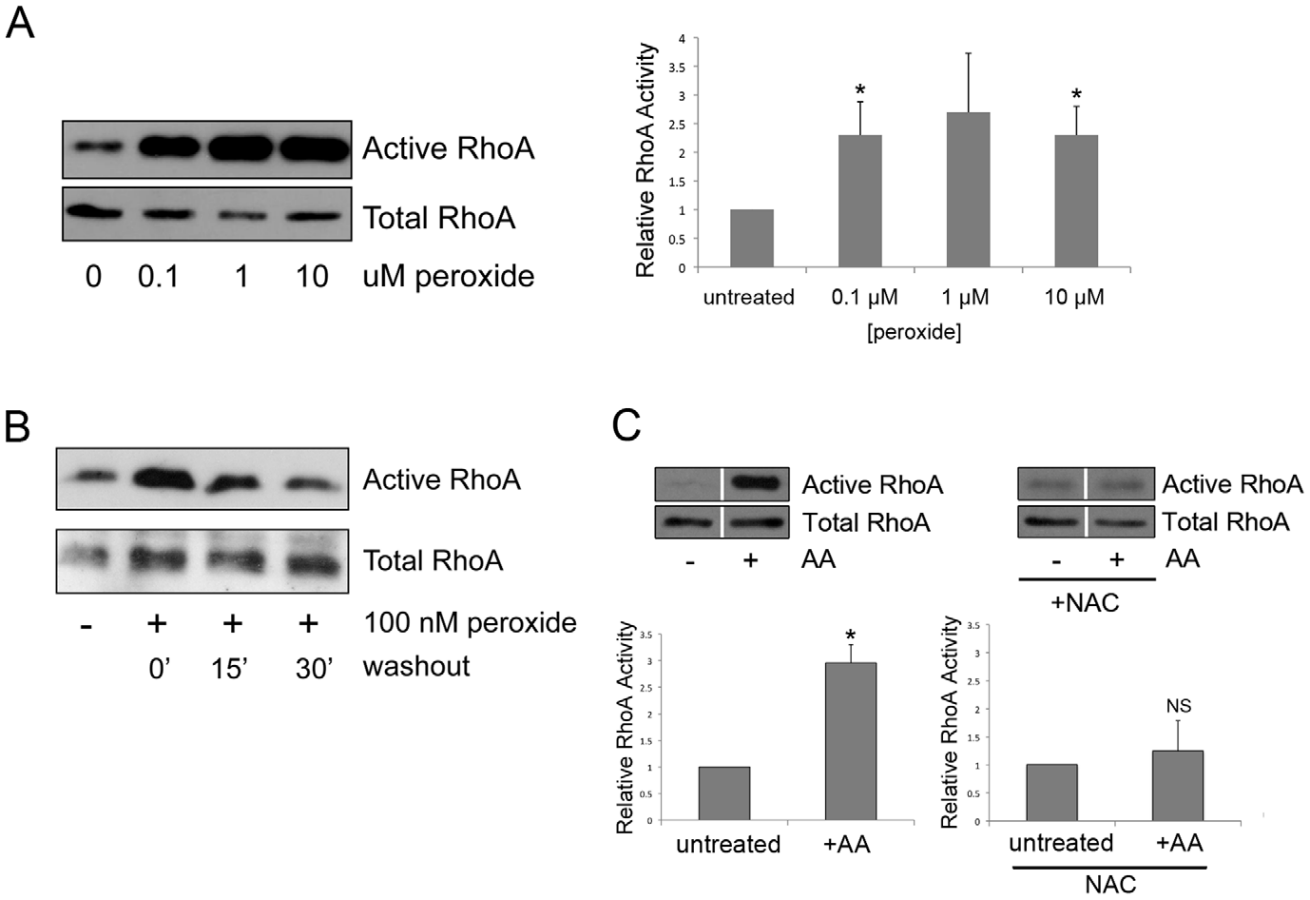
RhoA is reversibly activated upon exposure to ROS. (A) REF-52 fibroblasts were serum-starved followed by treatment with the indicated concentrations of t-butyl hydroperoxide (peroxide) in serum-free DMEM for 10 min. RhoA activity assays show that peroxide treatment results in the activation of endogenous RhoA. A representative blot of active RhoA sedimented by GST-RBD versus total RhoA from whole cell lysates is shown. ImageJ software was used to quantify RhoA activation. Relative RhoA activity is the ratio of active RhoA divided by total RhoA normalized to the untreated control. Graph represents the average +/− SEM of 5 independent experiments. * p<0.05. (B) REF-52 fibroblasts were serum-starved and treated as above with 100 nM peroxide for 10 min. Following peroxide treatment, the medium was either left unchanged (0′) or replaced with serum-free DMEM for 15 or 30 min (15′, 30′). The RhoA activity assay shows that peroxide-induced RhoA activation is reversible after washout. (C) REF-52 fibroblasts were serum-starved with or without 0.5 µM NAC followed by treatment with 10 µM antimycin A (AA) for 30 min. RhoA activity assays show that treatment with antimycin A results in the activation of endogenous RhoA. However, this activation is inhibited in cells pre-treated with NAC. Representative blots of active RhoA sedimented by GST-RBD versus total RhoA from whole cell lysates are shown. ImageJ software was used to quantify RhoA activation. Relative RhoA activity is the ratio of active RhoA divided by total RhoA normalized to the untreated control. Graph represents the average +/− SD of 3 independent experiments. * p<0.05, not signficiant (NS).

Peroxides are short-lived, membrane permeable oxidants that are generated *in vivo* by enzymatic complexes and as byproducts of cellular metabolism. To determine if exogenous ROS affects the activity of cellular RhoA, we treated REF-52 fibroblasts with low doses (0.1 µM to 10 µM) of a stable analog of hydrogen peroxide, t-butyl hydroperoxide (peroxide), for 10 min under serum-free conditions. The doses of exogenous peroxide used are physiologically relevant, based on measurements of intracellular ROS production made by others [6], [17]. Using standard Rhotekin-RBD pulldown assays, we observed that RhoA was significantly activated by peroxide treatment, as shown in Figure 1A and the accompanying quantification. To determine whether this activation is reversible, we treated REF-52 fibroblasts with 0.1 µM peroxide for 10 min, followed by washout with media only (Figure 1B). 0.1 µM peroxide activates endogenous RhoA, but the activity returns to baseline levels within 15–30 min upon washout. This reversibility suggests that the reducing environment of the cytoplasm and presence of intracellular anti-oxidants rapidly quench the effects of a bolus dose of exogenous peroxide. To determine whether endogenous ROS can activate RhoA, we used antimycin A, a compound that inhibits electron transport at complex III of the mitrochondrial respiratory chain, thereby inducing the production of superoxide and other ROS [18, 19, and Figure S1]. Treatment of cells with antimycin A resulted in activation of RhoA (Figure 1C). This activation was prevented by the presence of N-acetyl cysteine (NAC), a free radical and ROS scavenger. Having confirmed that endogenous ROS exerts the same effect on RhoA as extracellular peroxide, for all remaining experiments we use peroxide instead of antimycin A. We also examined whether peroxide would activate Rac1 in cells. With REF-52 fibroblasts, low levels of activation were detected in some experiments, but this was variable. However, with both HeLa cells (Figure S2) and endothelial cells (data not shown), we routinely observed robust activation of Rac1 in response to exogenous peroxide at 0.1 µM to 10 µM concentrations.

### ROS Treatment Induces Stress Fiber Formation

**Figure 2:**
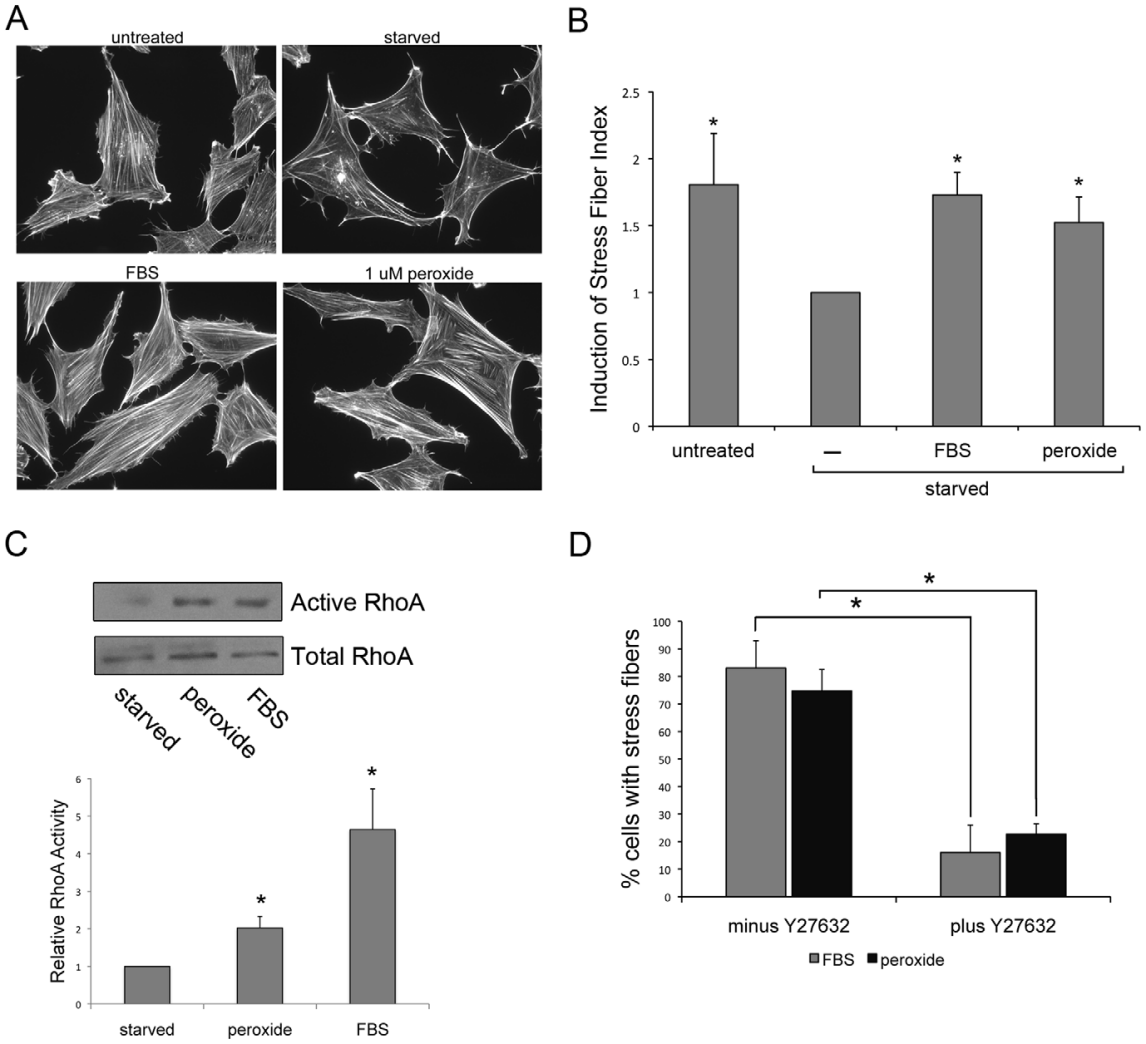
ROS treatment induces stress fiber formation. (A) Rat2 fibroblasts were grown on fibronectin-coated coverslips and either untreated, serum-starved for 1 h, or serum-starved followed by 15 min treatment with 5% FBS or 1 µM peroxide. F-actin staining was detected with Texas-Red labeled phalloidin. Serum-starvation results in loss of actin stress fibers; treatment with either FBS or peroxide induces prominent stress fibers. (B) Cells treated as above were scored for the presence of stress fibers and graphed as an index of stress fiber induction (normalized to serum-starved). Both FBS and peroxide result in an equally significant induction of stress fibers. Graph represents the mean of 3 independent experiments±SD, with >300 cells counted for each condition. Actual percent cells with stress fibers for each condition is 75.3±7.5% (untreated), 42.3 ± 4.6% (starved), 64.3±6.1% (serum-starved and peroxide treated), and 73±6.4% (serum-starved and FBS treated). * p<0.05. (C) Rat2 fibroblasts were serum-starved for 1 hr, followed by treatment with either 1 µM peroxide or 5% FBS for 15 min. RhoA activity assays show that both peroxide and FBS activate RhoA. A representative blot of active RhoA sedimented by GST-RBD versus total RhoA from whole cell lysates is shown. ImageJ software was used to quantify RhoA activation. Relative RhoA activity is the ratio of active RhoA divided by total RhoA normalized to the untreated control. Graph represents the average +/− SD of 2 independent experiments. * p<0.05. (D) Rat2 cells were grown as in panel A, serum-starved for 1 hr ±5 µM Y27632, and then treated for 15 min with 1 µM peroxide or 5% FBS. F-actin staining was detected with Texas-Red labeled phalloidin. Pre-treatment with Y27632 significantly reduces the percent of cells with prominent stress fibers after both peroxide and FBS treatment. Graph represents the mean of 3 independent experiments ± SD with >300 cells counted for each condition.

To further explore what functional consequences this ROS-mediated RhoA activation might have in a cellular context, we looked at stress fiber formation as it is a well-characterized cellular readout of Rho activity [2]. For these experiments, Rat2 cells were used because upon serum-starvation they readily lose stress fibers, allowing a low baseline from which induction of stress fibers could be induced and more readily quantified. Rat2 cells were serum-starved for 1 h to cause loss of stress fibers, and then treated with either 5% FBS as a positive control, or 1 µM peroxide for 15 min. Cells were fixed and F-actin was detected with Texas-Red labeled phalloidin. As expected, serum starvation results in significant loss of actin stress fibers in Rat2 fibroblasts (Figure 2A). Importantly, peroxide treatment induced formation of prominent stress fibers to a similar extent as adding back serum (Figure 2A). Stress fiber induction was quantified by scoring cells for the presence of stress fibers and is represented as an index of stress fiber induction in Figure 2B. The induction of stress fibers by peroxide and FBS correlated with activation of RhoA in Rat2 cells (Figure 2C). To further show the role of RhoA in the induction of stress fibers by peroxide and FBS, we used the Rho kinase inhibitor Y27632. Y27632 inhibits Rho kinase and therefore Rho-mediated contractility, preventing the formation of stress fibers [20]. Pre-treatment with Y27632 significantly reduced the percent of cells with prominent stress fibers after peroxide or FBS treatment (Figure 2D). The ability of ROS to induce cytoskeletal alterations has been described previously, particularly in the context of oxidant-mediated changes in endothelial cell permeability. These studies used both exogenous hydrogen peroxide [21], [22], [23], or superoxide and related molecules produced via the xanthine oxidase system [24]. It is important to note that these studies used peroxide concentrations in the range of 100–250 µM, and/or treatment for much longer time (1–6 hours) than we have utilized here (1 µM for 15 min). In addition to the formation of stress fibers, ROS treatment caused monolayer gap formation and increased permeability in these studies [22], [24], which is also indicative of elevated RhoA activity and increased actomyosin contractility induced via the Rho/Rho kinase signaling pathway. Consistent with these earlier reports, the data in Figure 1 and Figure 2 reveal that low concentrations of ROS activate RhoA and promote stress fiber formation in fibroblasts.

### Redox-Sensitive Cysteine Residues Are Required for ROS-Mediated Activation of RhoA

**Figure 3:**
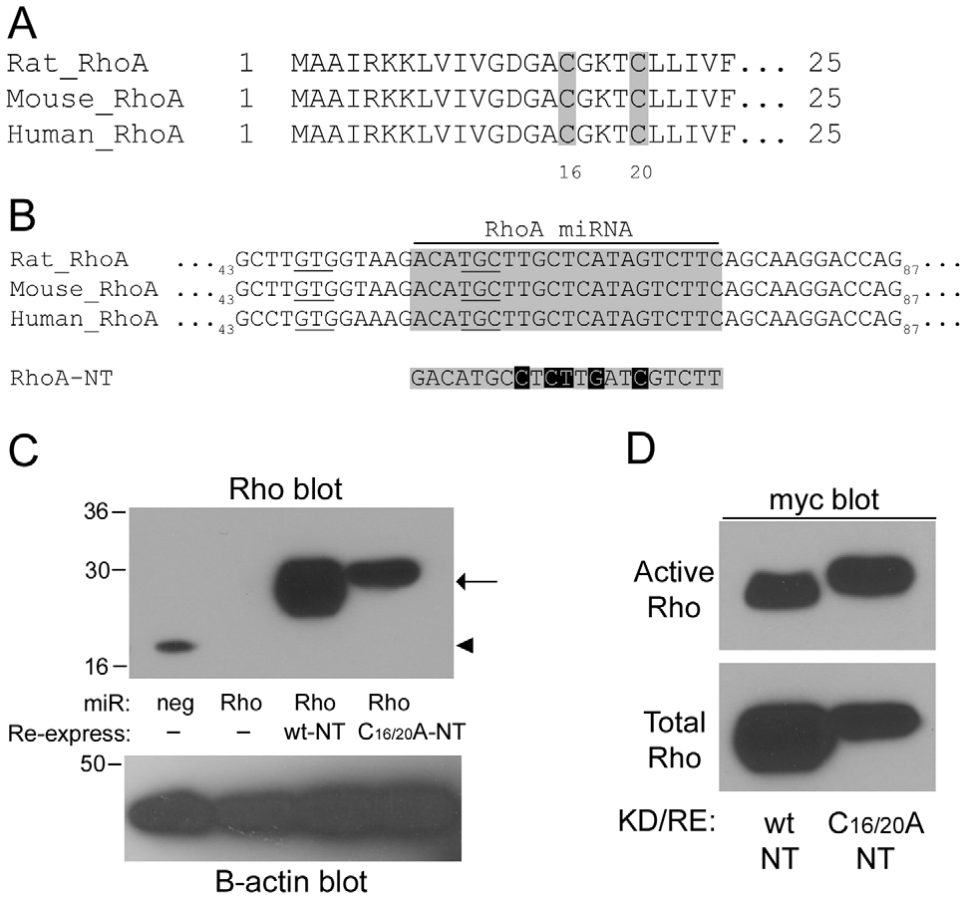
Characterization of RhoA miRNA and re-expression of non-targetable (NT) redox-resistant RhoA mutant constructs. (A) RhoA protein sequence (amino acids 1–25) showing conserved putative redox-sensitive cysteines C16 and C20 (shaded residues). (B) Nucleotide sequence (bp 43–87) of rat, mouse, and human RhoA showing the region targeted by the RhoA miRNA used in this study (shaded area). To re-express non-targeted RhoA constructs five silent mutations were introduced in the miRNA targeting region (black boxes). Nucleotides corresponding to C16 and C20 are underlined. (C) Knockdown of endogenous RhoA and simultaneous re-expression of non-targeted (NT) mutant constructs. REF-52 cells were infected with negative control or RhoA miRNA-encoding adenovirus together with virus encoding wt or C16/20A myc-RhoA where indicated. After 72 hours, total cell lysates were analyzed by western blot with a RhoA and B-actin antibody (loading control). Arrowhead indicates endogenous RhoA; arrow indicates the slower migrating non-targeting, myc-RhoA constructs. (D) Activity assay of wt and C16/20A myc-RhoA constructs. REF-52 cells were infected with negative control or RhoA miRNA-encoding adenovirus together with virus encoding wt or C16/20A myc-RhoA. After 72 hours, cells were processed for RhoA activity assays (in the presence of serum) and analyzed by western blot with a myc antibody.

What is the mechanism for this redox regulation of RhoA? Previously published *in vitro* observations have shown that RhoA has a redox-sensitive motif (GXXXCGK(S/T)C) containing two cysteine residues in the phosphoryl binding loop [16], [25]. As shown in Figure 3A, these cysteine residues are conserved in human, mouse, and rat RhoA. Previous *in vitro* studies revealed that oxidation of these cysteine residues, particularly cysteine 20, resulted in displacement of the bound nucleotide. Subsequent reduction allowed nucleotide to rebind, i.e. for nucleotide exchange to occur [16], [25]. To test whether these residues might play a role in the regulation of RhoA activity in cells by ROS, we mutated both cysteine 16 and 20 to alanine residues, thus making them resistant to oxidative modification. To prevent interference of endogenous RhoA in our experiments, we designed an adenovirally delivered miRNA construct targeted against a conserved region of RhoA, as shown in Figure 3B. Myc-tagged, non-targeted wildtype (wt) and C→A (C16/20A) mutants were designed with 5 silent mutations within the miRNA targeting sequence, thus allowing re-expression of the myc-tagged wt and C16/20A mutant proteins. Figure 3C demonstrates effective knockdown of endogenous RhoA and simultaneous re-expression of either wt or C16/20A myc-RhoA. Importantly, these myc-tagged, non-targeted RhoA constructs retain the ability to bind GTP as shown by GST-RBD pulldown assays in Figure 3D.

**Figure 4:**
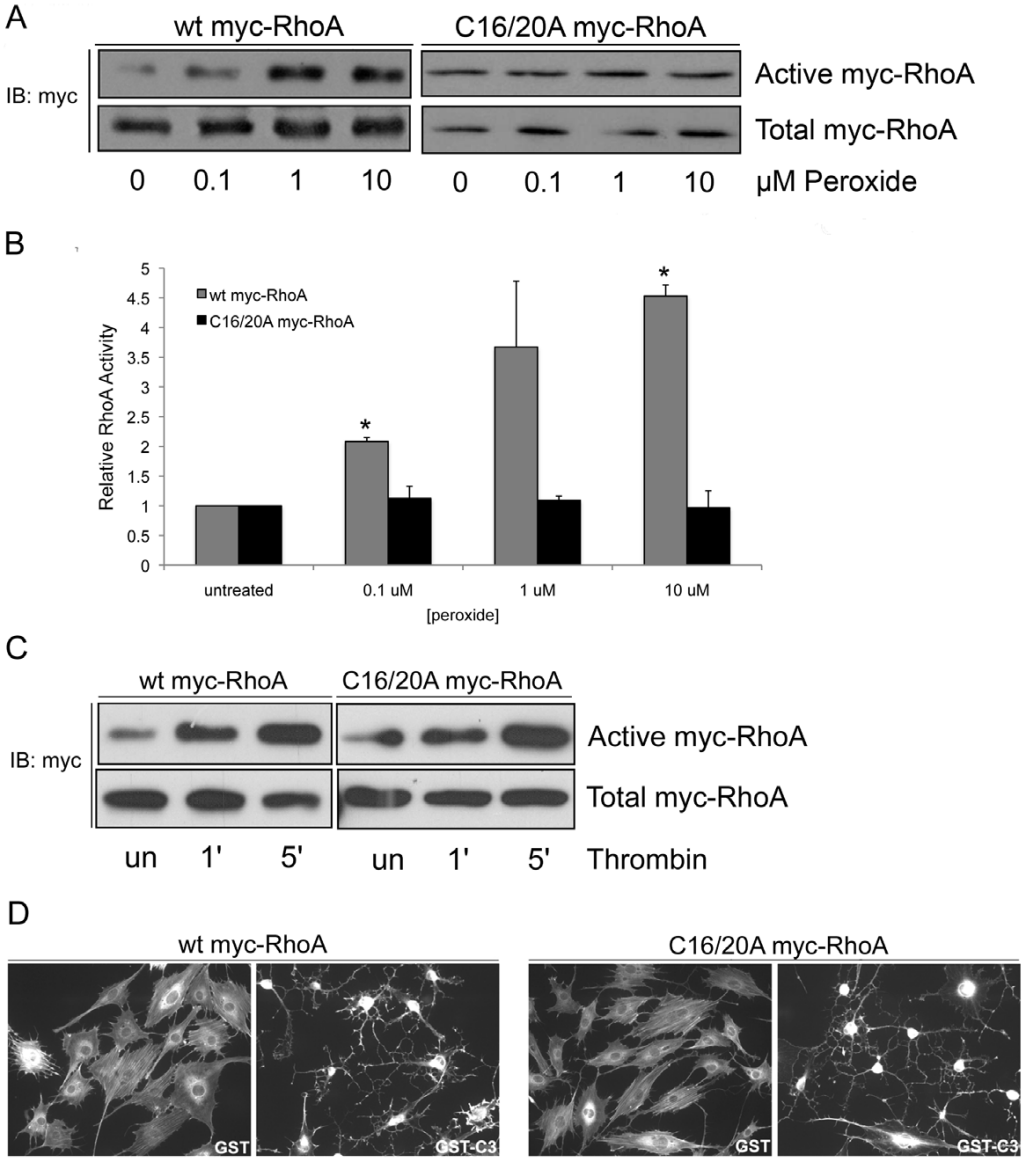
C16/20A myc-RhoA is not activated by ROS but responds normally to other physiological stimuli. (A) REF-52 cells expressing wt or C16/20A myc-RhoA were serum-starved and treated with the indicated concentrations of peroxide for 10 min. RhoA activity assays show that the activity of wt myc-RhoA increases in response to peroxide, however, the C16/20A mutant is resistant to activation by peroxide. (B) ImageJ software was used to quantify RhoA activation. Peroxide treated conditions are normalized to the corresponding untreated controls. Graph represents the average +/− SEM of 2 independent experiments. * p<0.05 compared to untreated control. (C) REF-52 cells in which endogenous RhoA had been knocked down and re-expressing wt or C16/20A myc-RhoA were serum starved and treated with 1 U/mL thrombin to test their responsiveness to GEF-mediated activation. RhoA activity assays show that both wt and C16/20A myc-RhoA are activated by thrombin at 1 and 5 min. (D) REF-52 cells in which endogenous RhoA had been knocked down and expressing wt or C16/20A myc-RhoA were treated with either GST alone or GST-C3, as described in the methods. F-actin staining was detected with Texas-Red labeled phalloidin. C3 treatment results in a characteristic dendritic morphology due to RhoA inactivation. Cells expressing either wt or C16/20A myc-RhoA develop the C3 phenotype.

To test whether cysteine 16 and 20 are required for the redox-sensitivity of RhoA, we examined the ability of C16/20A myc-RhoA to be activated by ROS in cells. REF52 cells expressing wt or C16/20A myc-RhoA were treated with various doses of peroxide under serum-free conditions, followed by RBD pulldown assay to detect RhoA activity. As shown in Figure 4A and the accompanying quantification (Figure 4B), while wt myc-RhoA responds to peroxide similarly to endogenous RhoA with activation over baseline levels, the C16/20A myc-RhoA mutant is resistant to activation by ROS. Importantly, data shown in Figure 4 indicate that while the C16/20A RhoA mutant is unresponsive to ROS treatment, it is still capable of being activated and inactivated by other physiological stimuli. Thrombin treatment is a well-characterized stimulus that induces RhoA activation in a wide variety of cell types [26], [27], an effect that is mediated through the action of p115 RhoGEF, among others [28], [29], [30]. Figure 4C shows that thrombin treatment activates wt RhoA as expected, and also rapidly activates C16/20A myc-RhoA, indicating that the mutant retains the ability to be activated by GEFs. Conversely, we tested the ability of C3 toxin to inactivate C16/20A myc-RhoA. C3 inactivates RhoA by ADP-ribosylation, and treatment of cells with this toxin results in a characteristic dendritic phenotype [31], [32]. REF52 cells expressing either wt or C16/20A myc-RhoA respond to C3 as indicated by this altered morphology (Figure 4D). Together, the results shown in Figure 4 indicate that the cysteine to alanine mutations confer resistance to ROS-mediated GTPase activation, but notably, still maintain responsiveness of the GTPase to other physiological stimuli.

### ROS-Mediated Stress Fiber Induction Requires Redox-Regulation of RhoA

**Figure 5:**
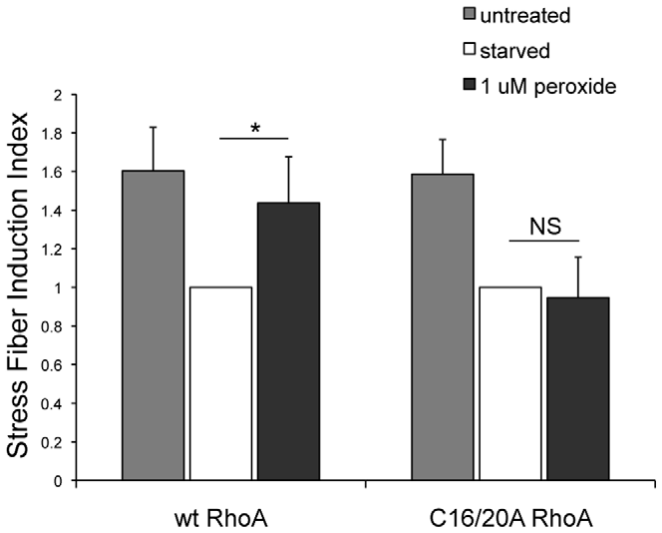
ROS induction of stress fibers is dependent on redox-regulation of RhoA. Rat2 fibroblasts were infected with adenoviruses to knockdown endogenous RhoA and re-express wt or C16/20A myc-RhoA. These cells were seeded on fibronectin-coated coverslips, and either left unstarved (DMEM +5% FBS), or serum-starved for 1 h followed by treatment with DMEM alone, or 1 µM peroxide in DMEM for 15 min. Quantitation of stress fiber induction shows that compared with the starved condition, peroxide treatment induces a statistically significant increase in stress fibers in cells expressing wt myc-RhoA but not in cells expressing redox-resistant C16/20A myc-RhoA. Graph represents the mean of 3 independent experiments, with >300 cells counted for each condition (error bars equal SD). * p<0.05; NS, not significant. Actual percent cells with stress fibers for each condition is: (unstarved) wt myc-RhoA 76.7±5.0%, C16/20A myc-RhoA 78.1±5.2%; (starved) wt myc-RhoA 48.3±5.7%, C16/20A myc-RhoA 49.6±5.0%; (peroxide) wt myc-RhoA 68.5±4.9%, C16/20A myc-RhoA 46.3±7.4%.

We went on to test how the C→A mutations affect the cellular function of RhoA by measuring peroxide-induced stress fiber induction in Rat2 cells as in Figure 2, but with cells re-expressing either wt or C16/20A myc-RhoA after knockdown of the endogenous protein. As shown in Figure 5, both wt and C16/20A myc-RhoA expressing cells have an equivalent amount of stress fibers in an unstarved state, and both lose stress fibers to a similar degree upon serum-starvation. However, while addition of 1 µM peroxide for 15 min induces stress fiber formation in cells re-expressing wt myc-RhoA cells expressing C16/20A myc-RhoA do not show any significant induction of stress fibers. These results confirm that cysteine 16 and/or 20 are specifically required for ROS-induced stress fiber formation by RhoA.

## Discussion

ROS and RNS have been implicated in a variety of cell signaling pathways, including growth factor signaling [6], [7], inflammation [33], engagement of integrins [34], [35], and adhesion to extracellular matrix (reviewed in [36]). Hydrogen peroxide in particular has gained considerable attention as a potent signaling molecule [37]. Hydrogen peroxide and other ROS have been shown to be generated both intracellularly and extracellularly. Extracellular ROS generated by neutrophils can act on endothelial cells to affect vascular permeability [38] and EGF receptor engagement has been shown to induce the production of extracellular peroxide which can then permeate the cell membrane to affect intracellular signaling [39], [40]. Most recently, the role of paracrine signaling by externally-produced peroxide has been shown in a zebrafish wound healing model. Niethammer et al. show that injured epithelial cells secrete peroxide (in the range of 0.5 to 50 uM) to induce leukocyte recruitment to the wound [41].

There is strong evidence that Rac1-derived ROS can lead to oxidative inactivation of LMW-PTP, resulting in phosphorylation and activation of p190RhoGAP and subsequent downregulation of RhoA [12]. However, several studies have demonstrated that exogenous peroxide induces effects consistent with activation of RhoA [21], [22], [23], [42]. Furthermore, Campbell et al. demonstrated *in vitro* that ROS and RNS induce nucleotide displacement by direct cysteine oxidation in the phosphoryl-binding site of RhoA. If this oxidative event is reversed by reduction and GTP is present in excess, nucleotide exchange (GTP binding) is favored and the GTPase is activated [25]. Notably, this occurs in the absence of GEFs. Our data support the mechanism described from their *in vitro* observations. We show that the activation of RhoA by peroxide is prevented by cysteine to alanine mutation of residues 16 and 20, although this mutant RhoA behaves normally in response to GEF-mediated situations (thrombin stimulation).

It is interesting that Zuckerbraun et al. found that treatment of cells with NO inhibits RhoA activity [43]. This apparent discrepancy has been observed in cell-based studies of the Ras GTPase, where both activation and inactivation of Ras have been observed in the presence of exogenously supplied RNS [44]. Redox regulation of Ras and Rho GTPase activity is likely to be dependent on a number of factors, including the type and level of cellular oxidants, reduction potential, as well as differences in cell lines resulting from variations in redox enzymes. If the oxidizing agent is in excess, or in the absence of a cellular reducing agent such as GSH, loss of GDP from RhoA can result in intramolecular disulfide formation between C16 and C20, and this form of RhoA is inactive, as it cannot bind nucleotide or interact with the GEF, Vav2, in *in vitro* studies [25]. However, under conditions where ROS/RNS are present at physiological levels and the reduction potential is high, as is the case within the cell cytoplasm, GTPase activation is expected, which is what we observe under our experimental conditions.

The cell's redox environment may also play a role in distinguishing regulation of RhoA by ROS as opposed to classical regulation by GEFs and GAPs. It is likely that ROS-mediated pathways are occurring parallel to pathways involving classical regulatory proteins. However, since ROS are typically very reactive and have short half-lives, direct regulation of RhoA by ROS is likely to be more spatially and temporally confined. This confinement may be relaxed under conditions where the cell's redox environment is out of balance. Certainly, there are situations where ROS are generated at increased levels physiologically and pathologically. For example, the respiratory system is normally exposed to greater concentrations of ROS than other tissues. Pulmonary diseases associated with airway hyperresponsiveness, such as asthma and COPD, have been shown to be associated with increased ROS production [45]. ROS and RhoA activation have been attributed to tracheal smooth muscle contractility, which is a hallmark of these disease states [46], [47]. We suspect that increased levels of ROS under these conditions may directly activate RhoA to drive smooth muscle contractility. Similarly, ischemia-reperfusion injury is associated with increased ROS production, which leads to pathology of the vascular system [48]. ROS-mediated activation of RhoA has been demonstrated in vascular smooth muscle [49]. Based on our results, we would predict that ROS may activate RhoA signaling in smooth muscle and in the endothelium, contributing to the massive increases in vascular permeability associated with ischemia-reperfusion injury.

In addition, we suspect that other Rho family GTPases may be directly regulated by ROS given the work of Campbell et al. [16]. For example, we show that Rac1 activity is regulated by peroxide in epithelial and endothelial cells (Supporting Figure S2 and data not shown). The regulation of Rac1 by ROS is particularly interesting because of its involvement in ROS production, through the NADPH oxidase complex [11], and its regulation by superoxide dismutase-1 (SOD1) [50]. SOD1 modulates intracellular ROS by converting superoxide to hydrogen peroxide. This creates a potential feedback loop in which Rac1 activity generates ROS which can regulate Rac1 activity through the redox-dependent sensor SOD1. Overall, these effects may be cell type specific, as the localization of a particular GTPase and baseline levels of activity likely play an important role in how that GTPase responds to ROS in terms of activity and phenotypic response.

We have shown that ROS can regulate RhoA activity in cells via a mechanism involving critical cysteine residues present in a redox-sensitive motif. This ROS-based mechanism of regulation was previously suggested by biochemical studies *in vitro* [25]. Our data support this occurring within cells and furthermore show that in a cellular context oxidative modification of these residues is a novel mechanism that can affect cytoskeletal dynamics.

## Materials and Methods

### Chemicals

T-butyl peroxide (peroxide) and N-acetyl cysteine (NAC) were purchased from Sigma-Aldrich (St. Louis, MO). Antimycin A was purchased from Axxora (San Diego, CA). Y-27632 was purchased from Calbiochem/EMD Chemicals (Gibbstown, NJ). 6-carboxy-2′,7′-dichlorodihydrofluorescein diacetate di(acetoxymethyl ester) (DCFDA) was purchased from Molecular Probes (Eugene, OR).

### Cell Lines

REF52 [51], Rat2 fibroblasts (CRL-1764, American Type Culture Collection), and HeLa cells (CCL-2, American Type Culture Collection) were grown in Dulbecco's modified Eagle's medium (DMEM; Gibco) supplemented with 10% fetal bovine serum (FBS; Sigma) and antibiotic-antimycotic solution (Gibco).

### Knockdown of RhoA Using miRNA Adenovirus

miRNA adenoviral constructs were designed and engineered using the BLOCK-iT ^TM^ Pol II miR RNAi expression vector system (Invitrogen) according to the manufacturer's protocol (Invitrogen). Briefly, double-stranded oligonucleotides were designed using Invitrogen's RNAi Designer (www.invitrogen.com/rnai) to form an engineered pre-miRNA sequence structure that targets a conserved region in human, mouse, and rat RhoA:

5′TGCTGAAGACTATGAGCAAGCATGTCGTTTTGGCCACTGACTGACGACATGCTCTCATAGTCTT3′ (21 bp antisense target sequence underlined). Synthesized oligonucleotides were annealed and ligated into pcDNA 6.2-GW/EmGFP-miR. The EmGFP-miRNA cassette was subsequently shuttled through pDONR221(Invitrogen) by Gateway BP recombination and then into pAd-CMV-Dest Gateway vector by LR recombination. Each construct was sequence verified and virus was produced in 293A packaging cell line with the ViraPower Adenoviral Expression System (Invitrogen) using the manufacturer's recommended protocol.

### Generation of Non-Targeting RhoA Constructs and Adenoviral-Mediated Expression

In order to simultaneously knockdown endogenous RhoA while allowing re-expression of RhoA mutants, non-targeting RhoA constructs were designed. Human RhoA in pCMV-myc was used as a template for site-directed mutagenesis (QuikChange II Site-Directed Mutagenesis kit; Stratagene), wherein 5 silent DNA basepair mutations were made within the miRNA targeting region of RhoA. This non-targeted form of RhoA was then used as a template for site-directed mutagenesis in order to generate cysteine to alanine mutants at C16 and C20 (C16/20A myc-RhoA). Adenovirus was generated using the Virapower adenoviral expression system (Invitrogen) following the manufacturer's protocol as described above. For experiments, cells were infected by overnight incubation with adenovirus-containing media followed by addition of fresh DMEM/10% FBS for another 48 h.

### Immunohistochemistry and Measurement of Stress Fiber Induction

Rat2 cells treated with adenovirus to knockdown endogenous RhoA while re-expressing either wt myc-RhoA or C16/20A myc-RhoA were plated onto fibronectin-coated coverslips (15 ug/ml) overnight, followed by serum-starvation for 1 h. Cells were treated for 15 min with either serum-free or 1 µM peroxide-containing DMEM, or 5% FBS-containing DMEM (positive control) before fixing/permeabilizing in 3.7% paraformaldehyde, then 0.02% Triton X-100. In some experiments, Rat2 cells were starved in the presence of 5 µM Y27632. Texas Red phalloidin (Molecular Probes) was used to stain F-actin. Immunofluorescence images were taken through a 20x objective (Zeiss plan-Apochromat 20x/0.8) with a Zeiss axiovert 200 M microscope equipped with a Hamamatsu ORCA-ERAG digital camera and acquired using Metamorph Workstation (Universal Imaging Corp.). To quantify stress fiber induction, GFP-positive cells were scored by a blinded observer for the presence or absence of stress fibers; the criteria were: organized, thickened parallel actin bundles throughout the majority of the cytoplasm. Results are plotted as a “stress fiber induction index” which was obtained by normalizing each treatment to the starved condition. Statistical significance was determined by Student's t-test of the average from 3 independent experiments.

### RhoA Activity Assays

RhoA activity assays [52] were performed as described previously with minor modifications [53]. Cells were serum-starved in DMEM for 2 hours prior to performing assays to lower baseline RhoA activity and remove serum proteins which would interfere with experimental conditions. Cells were washed with ice-cold HEPES buffered saline (pH 7.4) and lysed in Buffer A (50 mM Tris pH 7.6, 500 mM NaCl, 1% Triton X-100, 0.1% SDS, 0.5% deoxycholate, 10 mM MgCl_2_, 100 µM orthovanadate and protease inhibitors). Lysates were clarified by centrifugation and equalized for total volume and protein concentration. After incubation with glutathione S-transferase (GST)-Rho binding domain (RBD) beads, washing three times with ice-cold Buffer B (50 mM Tris pH 7.6, 150 mM NaCl, 1% Triton X-100, 0.5 mM MgCl_2_, 100 µM orthovanadate, with protease inhibitors, the bound fraction (active GTP-RhoA) was analyzed by SDS-PAGE. Total RhoA levels were similarly analyzed using a reserved aliquot of whole cell lysate. Active RhoA and total RhoA were analyzed by Western blotting with an anti-RhoA antibody (monoclonal antibody 26C4; Santa Cruz Biotechnology, Santa Cruz, CA). The results were quantified using ImageJ software (NIH; Bethesda, MD). The relative amount of active RhoA was determined by taking the ratio of RhoA sedimented by GST-RBD beads (active RhoA) divided by the amount of total RhoA in the whole cell lysate. Statistical significance was determined using Student t-test (one-tailed).

### Rac1 Activity Assays

Rac1 activity assays [54], [55] were performed as described previously with minor modifications [56]. Cells were serum-starved in DMEM for 1 hour prior to performing assays to lower baseline Rac1 activity and to remove serum proteins which would interfere with experimental treatments. Cells were washed with ice-cold HEPES buffered saline (pH 7.4) and lysed in Buffer B (50 mM Tris pH 7.6, 150 mM NaCl, 1% Triton X-100, 0.5 mM MgCl_2_, 100 µM orthovanadate, and protease inhibitors). Lysates were clarified by centrifugation and equalized for total volume and protein concentration. After incubation with glutathione S-transferase (GST)-PAK binding domain (PBD) beads and washing three times with ice-cold Buffer B, the bound fraction (active GTP-Rac1) was analyzed by SDS-PAGE. Total Rac1 levels were similarly analyzed using a reserved aliquot of whole cell lysate. Active Rac1 and total Rac1 were analyzed by Western blotting with a monoclonal anti-Rac1 antibody clone 102 (BD Transduction Laboratories, San Jose, CA). The results were quantified using ImageJ software (NIH; Bethesda, MD). The relative amount of active Rac1 was determined by taking the ratio of Rac1 sedimented by GST-PBD beads (active Rac1) divided by the amount of total Rac1 in the whole cell lysate. Statistical significance was determined using Student t-test (one-tailed).

### Measurement of ROS Generation

Formation of ROS was monitored by the conversion of non-fluorescent 6-carboxy-2′,7′-dichlorodihydrofluorescein diacetate di(acetoxymethyl ester) to fluorescent DCF. Cells were loaded with 5 µM DCF in serum-free DMEM for 30 min at 37°C. After loading, cells were washed twice with phosphate-buffered saline, and incubated for an additional 20 min at 37°C to allow for dye de-esterification. Cells were stimulated with antimycin A or peroxide as described in the figure legends. Fluorescence was determined using a fluorometer with an excitation of 485 and an emission of 520.

### C3 Treatment

GST-tagged recombinant proteins (either GST alone or GST-C3) were isolated from BL-21 bacteria using the manufacturer's protocol for GST fusion proteins (Amersham Pharmacia Biotech). Following adenovirus infection of REF-52 cells to knockdown endogenous and re-express wt or C16/20A myc-RhoA as described above, C3 toxin was introduced into these cells on coverslipsusing Lipofectamine according to the protocol of Maddox et al. [57]. After 3 hours of C3 exposure, cells were fixed and imaged as described above.

## Supporting Information

Figure S1 Antimycin A induces ROS production. REF52 fibroblasts were loaded with 5 µM dichlorofluorescin diacetate (DCFDA) for 30 min under serum free conditions, followed by stimulation with 10 µM antimycin A (AA) for 5, 15 or 30 min. Stimulation with 10 µM peroxide for 5 min was used as a positive control.
(0.04 MB TIF)

Figure S2 Peroxide activates Rac1 in HeLa Cells. HeLa cells were serum-starved and treated with the indicated concentrations of peroxide for 10 min. Rac1 activity assays show that peroxide treatment results in activation of endogenous Rac1. A representative blot of active Rac1 sedimented by GST-PBD versus total Rac1 from whole cell lysates is shown. ImageJ software was used to quantify Rac1 activation. Graph represents the average +/− SD of 2 independent experiments. * p<.05 versus untreated control.
(0.12 MB TIF)
