# Supplementary figures and images for: Direct Activation of RhoA by Reactive Oxygen Species Requires a Redox-Sensitive Motif
Source: PLoS One. 2009 Nov 26;4(11):e8045. doi: 10.1371/journal.pone.0008045 (PMC2778012; doi:10.1371/journal.pone.0008045)

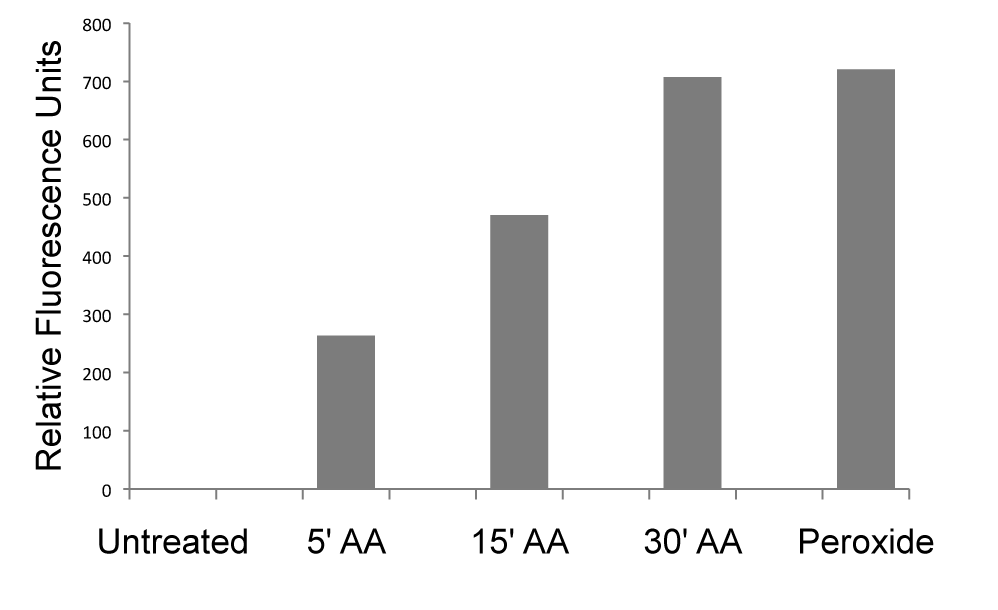

Supplement: Figure S1 — Antimycin A induces ROS production. REF52 fibroblasts were loaded with 5 µM dichlorofluorescin diacetate (DCFDA) for 30 min under serum free conditions, followed by stimulation with 10 µM antimycin A (AA) for 5, 15 or 30 min. Stimulation with 10 µM peroxide for 5 min was used as a positive control. (0.04 MB TIF) [file pone.0008045.s001.tif]

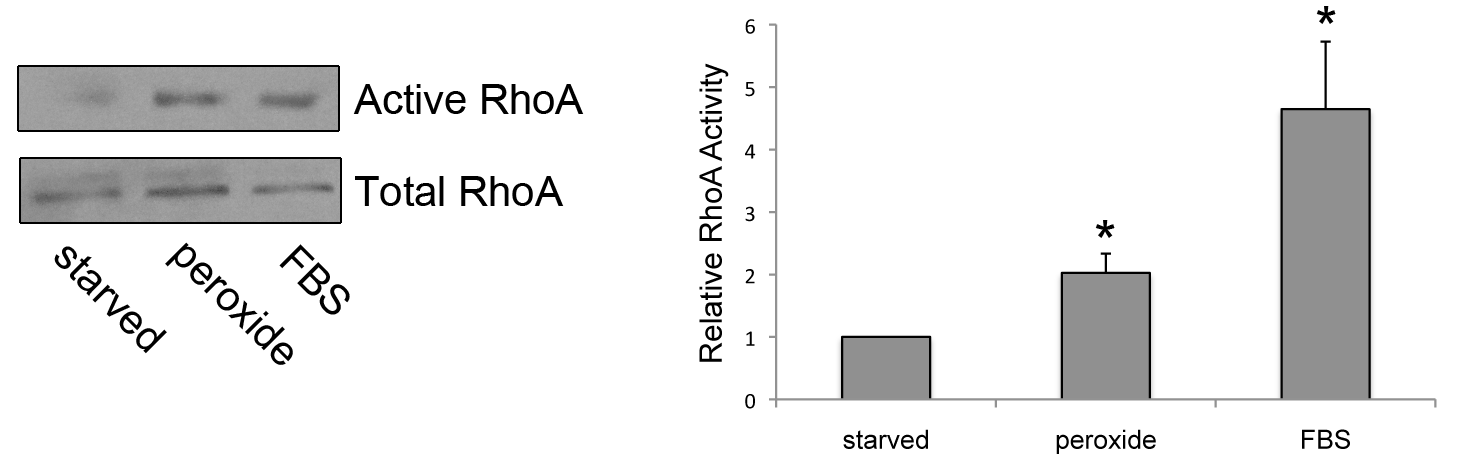

Supplement: Figure S2 — Peroxide activates Rac1 in HeLa Cells. HeLa cells were serum-starved and treated with the indicated concentrations of peroxide for 10 min. Rac1 activity assays show that peroxide treatment results in activation of endogenous Rac1. A representative blot of active Rac1 sedimented by GST-PBD versus total Rac1 from whole cell lysates is shown. ImageJ software was used to quantify Rac1 activation. Graph represents the average +/− SD of 2 independent experiments. * p<.05 versus untreated control. (0.12 MB TIF) [file pone.0008045.s002.tif]
